# Caffeine as a Potential Quorum Sensing Inhibitor

**DOI:** 10.3390/s130405117

**Published:** 2013-04-18

**Authors:** Siti Nur Maisarah Norizan, Wai-Fong Yin, Kok-Gan Chan

**Affiliations:** Division of Genetics and Molecular Biology, Institute of Biological Sciences, Faculty of Science, University of Malaya, Kuala Lumpur 50603, Malaysia; E-Mails: maisarahnorizan@siswa.um.edu.my (S.N.M.N.); wfyin@um.edu.my (W.F.Y.)

**Keywords:** AHL synthesis inhibition, anti-infective drugs, *Chromobacterium violaceum* CV026, *N*-acyl-L-homoserine lactones (AHL), *Pseudomonas aeruginosa* PA01, swarming, quorum sensing inhibitor, virulence

## Abstract

Quorum sensing enables bacteria to control the gene expression in response to the cell density. It regulates a variety of bacterial physiological functions such as biofilm formation, bioluminescence, virulence factors and swarming which has been shown contribute to bacterial pathogenesis. The use of quorum sensing inhibitor would be of particular interest in treating bacterial pathogenicity and infections. In this work, we have tested caffeine as quorum sensing inhibitor by using *Chromobacterium violaceum* CV026 as a biosensor. We verified that caffeine did not degrade the *N*-acyl homoserine lactones tested. In this work, it is shown that caffeine could inhibit *N*-acyl homoserine lactone production and swarming of a human opportunistic pathogen, namely *Pseudomonas aeruginosa* PA01. To the best of our knowledge, this is the first documentation providing evidence on the presence of anti-quorum sensing activity in caffeine. Our work will allow caffeine to be explored as anti-infective drugs.

## 1. Introduction

Bacteria have developed a form of cell-cell communication system that allows them to communicate. This system is called the quorum sensing (QS), whereby communication within the bacteria involves the production and sensing of small diffusible signal molecules produce by the bacteria. QS was first found in the marine bioluminescent bacterium *Vibrio fischeri* [1–4]. When threshold level of the signal molecules has been reached, it will mediate bacteria regulation feedback regulatory loop [5,6]. Consequently, QS controlled various phenotypes such as biofilm formation [7–10], bioluminescence [1–4], virulence factors [11] and swarming [12,13] which has been shown contribute to bacterial pathogenesis.

There are three QS systems in bacteria which are: (1) LuxI/LuxR-type QS in proteobacteria which use *N*-acylhomoserine lactones (AHL) as signaling molecules; (2) luxS-encoded autoinducer 2 (AI-2) system that exist in both Gram-positive and Gram-negative bacteria; (3) oligopeptide-two-component-type QS in Gram-positive bacteria [13,14]. As the pathogenicity traits of bacteria are controlled by QS, therefore anti-QS is an alternative measure in combating bacterial pathogenicity. This alternative treatment which doesn't rely on antibiotics and prevent drug-resistance problem is highly desirable.

To date, many studies have been conducted in finding new anti-QS compounds that are analogs to the naturally occurring AHL signals molecules and inhibit the AHL signal receptor proteins. Among the few non bacterial-origin antagonists of QS that have been found are catechin (from *Combretum albiflorum* bark extract) [15], halogenated furanones (from red alga *Delisea pulchra*) [16], Malabaricone C (from *Myristica cinnamomea*) [17], and also the extract of vanilla [18], *Syzygium aromaticum* [19], and *Melicope lunu-ankenda* [20]. However, no study has been conducted to investigate the potential of caffeine as an anti-QS compound.

Caffeine (1,3,7-trimethylxanthine) is one of the few plant products with which the general public is readily familiar, because of its widespread occurrence in beverages such as coffee, tea and various soft drinks [21]. It is classified as an alkaloid, and has a white crystalline appearance and bitter taste. This famous stimulant drug naturally acts as a pesticide that paralyzes and kills certain insects, larvae and beetles [22] that feed on plants. The identity of caffeine remained a mystery until the year 1820 when a German chemist, Friedlieb Runge, managed to isolate pure caffeine for the first time and called it “Kaffebase” meaning a base that exist in coffee. Previous studies have shown that caffeine at a concentration of 2.5 mg/mL retards the growth of *Escherichia coli*, *Enterobacter aerogenes*, *Proteus vulgaris*, and *Pseudomonas aeruginosa* within a short time [23]. In addition, it is also reported that the concomitant use of caffeine with amoxicillin potentiates the antibacterial effect of amoxicillin against *Staphylococcus aureus* [24]. Hence, this work aimed to study the potential of caffeine as a potential anti-QS compound.

## 2. Experimental Section

### 2.1. Sample Preparation

Caffeine was obtained from Sigma (St. Louis, MO, USA). Caffeine samples were prepared fresh by dissolving caffeine powders in ultrapure water at 80 °C. The aliquots were then sterilized by filtration (using 0.25 μm filter pore size) and diluted to appropriate concentrations using sterilized ultrapure water. (+)-Catechin was purchased from Sigma and dissolved in 20% DMSO before sterilized by filtration (using 0.25 μm filter pore size) and further diluted to appropriate concentrations using sterilized ultrapure water.

### 2.2. Bacterial Strains and Culture Condition

*Chromobacterium violaceum* CV026 (Mini-Tn*5* mutant derived from *C. violaceum* ATCC 31532 Hg^R^, *cvil*::Tn*5 xyl*E, Kan^R^, plus spontaneous Str^R^) was used in this study [25]. *C. violaceum* CV026 is an AHL biosensor that will produce a purple pigment in the presence of s short chain AHL. CV026 was cultured in Luria-Bertani (LB) broth (1% w/v peptone, 0.5% w/v yeast extract, 0.5% w/v NaCl, per 100 mL distilled water) buffered with 50 mM 3-(*N*-morpholino)propanesulfonic acid (MOPS) to pH 6.8. *C. violaceum* CV026 culture was incubated at 28 °C with shaking (220 rpm) while *Pseudomonas aeruginosa* PA01 was routinely cultured at 37 °C.

### 2.3. Antibacterial Assay

A paper disc diffusion assay to assess the antibacterial activity of caffeine was performed according to the Clinical and Laboratory Standard Method with certain modifications [26]. *C. violaceum* CV026 was incubated for 16–18 hours before the OD_600nm_ was adjusted to 0.1. 100 μL of the bacteria culture were then spread on the MHA plates and left to dry for 30 minutes. Paper disc (10 mm diameter) were placed on the plates and 20 μL of caffeine dissolved in ultrapure water at final concentration of 0.1, 0.2, 0.3 and 0.4 mg/mL were loaded on the discs. Ultrapure water, 20% DMSO and 20 μL of (+)-catechin dissolved in 20% DMSO at final concentration of 0.1, 0.2, 0.3 and 0.4 mg/mL were also included as negative controls. DMSO (100%) was used as positive control. The plates were incubated at 28 °C for 24 hours and observed for any growth and inhibition zones. This assay was repeated in three independent triplicates.

### 2.4. Screening of Caffeine for Anti-QS Properties

This assay was conducted as described by Krishnan *et al.* [19] with modifications. Briefly, *C. violaceum* CV026 lawn was prepared by adding 8 mL of overnight culture adjusted to OD_600nm_ of 1.2 into 40 mL of warm molten LB agar buffered with 50mM MOPS. *N*-hexanoylhomoserine lactone (C6-HSL) was also added into the agar mixture at the final concentration of 0.12 μg/mL. The agar was gently mixed and poured immediately into a Petri dish (150 mm diameter) and left to solidify for 2 hours. Wells (4 mm diameter) were made on the solidified agar plate. Caffeine with final concentration of 0.1, 0.2 and 0.3 mg/mL were loaded into each well (40 μL per well). (+)-catechin was used as positive control for QS inhibition while ultrapure water and DMSO served as negative controls. The plate was incubated for 18–24 hours at 28 °C to check for the violacein inhibition. Halo zone formation on the purple background suggested that caffeine exhibited anti-QS property. This assay was repeated in three independent triplicates.

### 2.5. Anti-QS Activity of Caffeine against C. Violaceum CV026

*C. violaceum* CV026 overnight culture grown in LB broth was adjusted to an OD600 of 0.01 followed by the addition of C6-HSL to a final concentration of 0.12 μg/mL. Then, 10 mL of the aliquots were transferred into 50-mL sterile plastic tubes followed by addition of caffeine solution to a final concentration of 0.1, 0.2, and 0.3 mg/mL, respectively. Ultrapure water was included as negative control. The mixture was vortex for 5 seconds and incubated for 16–18 hours at 28 °C with gentle shaking. Bacterial cultured without addition of C6-HSL but treated with caffeine (same concentrations and treated as described above) was prepared. After 16–18 hours incubation, the tubes were then vortex for 30 seconds to re-suspend any pellicle of adherent cells. Then, 200 μL of bacterial culture with various treatments were then added into 96-well flat-bottomed microplate (SPL Life sciences) and violacein concentration was monitored at 585 nm. Experiments were done in triplicate. The optical density of the culture was read using an Infinite M200 luminometer (Tecan, Mannerdorf, Switzerland) at wavelength 600 nm.

### 2.6. Quantification of Violacein

The production of violacein was quantified as previously described [18]. In summary, tubes containing overnight culture and treatment were vortex for 30 seconds to re-suspend any pellicle of adherent cells after 16–18 hours incubation. Bacterial culture (1 mL) of each tube was centrifuged at 13,000 rpm for 10 minutes to precipitate the insoluble violacein. The cultures supernatant was discarded and 1 mL of 100% DMSO was added to the pellet. The solution was then vortex vigorously for 30 seconds to ensure that the violacein has completely solubilized and centrifuged at 13,000 rpm for 10 minutes to remove the cells. Then, 200 μL of the violacein-containing supernatants were then added into 96-well flat-bottomed microplate (SPL Life Sciences, Pocheon-Si, Korea) in triplicate. The absorbance was read using the Tecan Infinite M200 luminometer at a wavelength of 585 nm.

### 2.7. Screening of N-Hexanoyl Homoserine Lactone Degradation by Caffeine

This assay was performed as described by Chan *et al.* [27] with modifications. Briefly, caffeine was added at final concentration of 0.1, 0.2, and 0.3 mg/mL into 2 mL microcentrifuge tubes filled with 1 mL of LB broth with 50 mM MOPS and C6-HSL (0.12 μg/mL). The tubes were incubated overnight at 28 °C with shaking (220 rpm). After 16–18 hours incubation, extraction of C6-HSL from the mixture was conducted by adding equal volume of ethyl acetate into each tubes and vortexed vigorously. After which, the supernatant (containing ethyl acetate with C6-HSL) was transferred into new tubes and air-dried in a fume hood. After drying, 0.1 mL of phosphate buffered saline (PBS) (10 mM, pH 7.4) was added into each microcentrifuge tubes to rehydrate C6-HSL. For the detection of C6-HSL degradation, 10 μL of the mixture was spotted onto sterile paper discs placed on CV026 lawn and incubated overnight at 28 °C. Decreased violacein production (purple zone) indicated C6-HSL degradation. This assay was repeated in three independent triplicates.

### 2.8. Inhibition of AHLs Synthesis by Pseudomonas aeruginosa PA01

Briefly, 250 μL of caffeine at final concentration of 0.1 to 1.0 mg/mL were added into 15 mL of *P. aeruginosa* PA01 overnight culture (OD_600nm_ of 0.1) grown in LB broth buffered with 50 mM MOPS. The mixture was vortex gently before incubated for 16–18 hours at 37 °C (220 rpm). After 16–18 hours incubation, the tubes were vortex for 30 seconds to re-suspend any pellicle of adherent cells. Bacterial culture (200 μL) were added into each well of the 96-well flat-bottomed microplate (SPL Life Sciences) in triplicate. The absorbance of each well was read using the Tecan Infinite M200 luminometer at a wavelength of 600 nm. Extraction of the short chain AHLs from the remaining overnight culture was conducted after that as explained in previous section. The extracted AHLs (20 μL) was spotted onto sterile paper discs placed on CV026 lawn and incubated overnight at 28 °C for 24 hours. Decreased violacein production (purple zone) indicated the inhibition of *Pseudomonas aeruginosa* PA01 QS signal. This assay was repeated in three independent triplicates.

### 2.9. Bacterial Growth

*P. aeruginosa* PA01 growth was studied using methods reported by Hayouni and colleagues [28] with modifications. Briefly, overnight cultures of *P. aeruginosa* PA01, were diluted to OD_600nm_ of 0.1. The overnight bacterial culture and appropriate concentration of caffeine were placed in a 96-well flat-bottomed microplate (SPL Life Sciences) at a final volume of 200 μL in each well. The optical density OD_600nm_ were determined every 30 minutes for 24 hours using the Tecan Infinite M200 luminometer. The growth of *P. aeruginosa* PA01 was determined by plotting the OD_600nm_ against time.

### 2.10. Inhibition of Pseudomonas aeruginosa PA01 Swarming Motility

To verify application of caffeine to inhibit QS-mediated virulence, we used swarming assay using *P. aeruginosa* PA01 as selected pathogen. Swarming agar was freshly prepared using the following composition: glucose (1% w/v), Bacto agar (0.5% w/v), Bacto peptone (0.5% w/v) and yeast extract (0.2% w/v) [29]. Then, 250 μL of caffeine at final concentration of 0.1 mg/mL, 0.2 mg/mL and 0.3 mg/mL were seeded into 14.75 mL of the molten swarming agar, mixed well gently and dispensed onto Petri dishes. Then, 2 μL of *P. aeruginosa* PA01 overnight culture (OD_600nm_ of 0.1) were inoculated at the centre of the agar and incubated for 16 hours at 37 °C. Reduction in *P. aeruginosa* PA01 swarming motility indicates anti-QS properties of caffeine. This assay was repeated in three independent triplicates.

### 2.11. Statistical Analysis

All statistical results in this study represent the average of three independent experiments. The data were analyzed using one-way ANOVA test at P < 0.05 using GraphPad Prism 5 statistical software.

## 3. Results and Discussion

### 3.1. Antibacterial Assay

**Figure 1.**
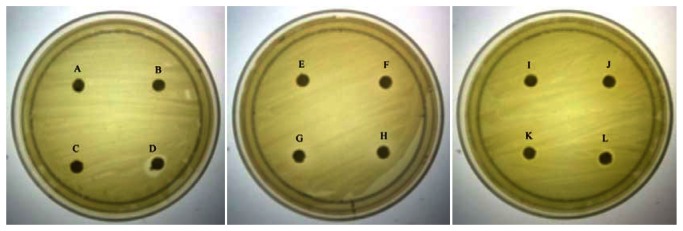
Antibacterial assay. (**A**) Disc without treatment; (**B**) 20% DMSO; (**C**) Ultrapure water; (**D**) 100% DMSO; (**E**) 0.1 mg/mL caffeine; (**F**) 0.2 mg/mL caffeine; (**G**) 0.3 mg/mL caffeine; (**H**) 0.4 mg/mL caffeine; (**I**) 0.1 mg/mL; (**J**) 0.2 mg/mL (+)-catechin; (**K**) 0.3 mg/mL (+)-catechin; (**L**) 0.4 mg/mL (+)-catechin. None of the tested caffeine concentrations showed growth inhibition zones except for paper disc treated with 100% DMSO (positive control). This assay was conducted in three independent triplicates.

As QS inhibition is focused on the interference of bacterial signaling and not antibacterial activity, it is important to ensure any anti-QS effect is not resulted from antibacterial activity. Therefore, paper disc diffusion assay was performed to test antibacterial activity of caffeine against *C. violaceum* CV026. Our result (Figure 1) shown that there was no inhibition zones observed suggesting all tested concentrations of caffeine showed no antibacterial activity. This indicates that caffeine at these concentrations does not inhibit the growth of *C. violaceum* CV026. Visible inhibition zone was only observed on the disc treated with 100% DMSO (positive control).

### 3.2. Screening of Caffeine for Anti-QS Properties

**Figure 2.**
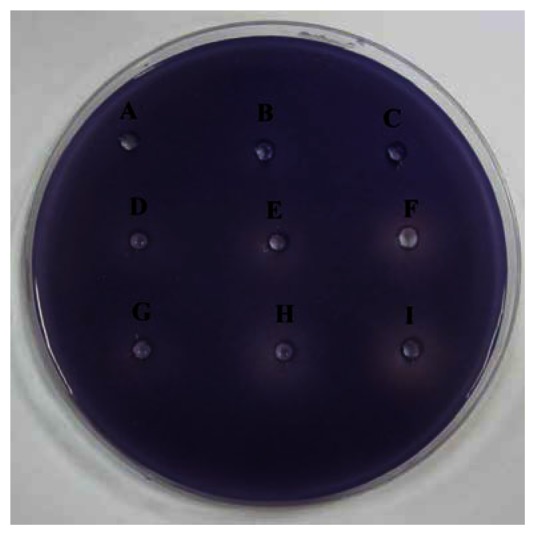
Anti-QS properties of caffeine. (**A**) untreated well; (**B**) 20% DMSO; (**C**) ultrapure water; (**D**) 0.1 mg/mL (+)-catechin; (**E**) 0. 2 mg/mL (+)-catechin; (**F**) 0.3 mg/mL (+)-catechin; (**G**) 0.1 mg/mL caffeine; (**H**) 0.2 mg/mL caffeine; (**I**) 0.3 mg/mL caffeine. Result showed that caffeine promoted QS inhibitory effect in concentration dependent manner. This assay was conducted in three independent triplicates.

The preliminary screening of anti-QS properties of caffeine was done by using *C. violaceum* CV026 as biosensor. Our results have shown that the increased amount of caffeine shown increased inhibition suggesting that higher the concentration of caffeine, the stronger inhibition of *C. violaceum* CV026 responding to C6-HSL (Figure 2).

### 3.3. Caffeine Shown Anti-QS Properties but not Antibacterial Activity Using C. Violaceum CV026 Bioassay

**Figure 3.**
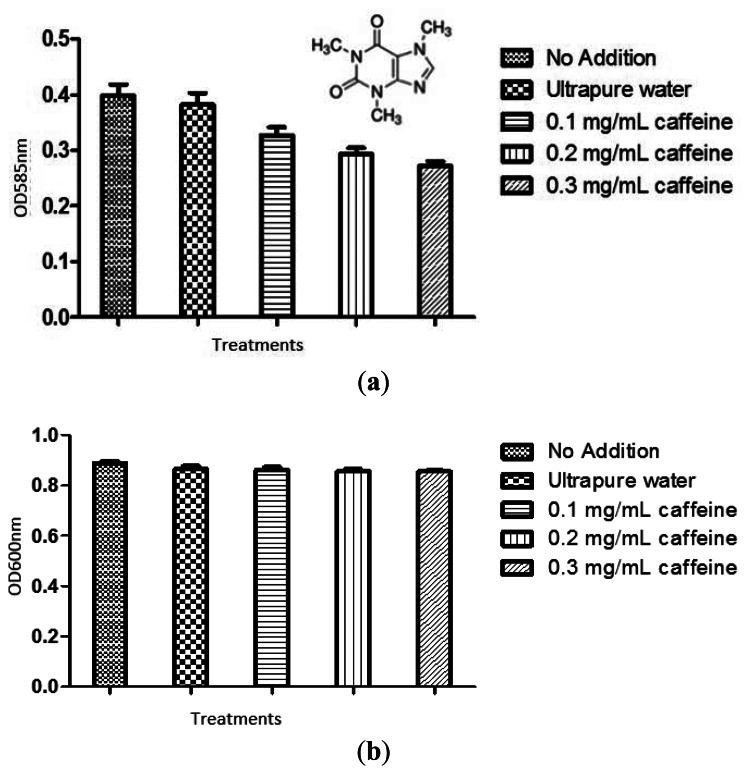
Caffeine inhibits CV026 violacein production by anti-QS. The violacein production was measured spectrophotometrically as described in Materials and Methods and quantified by reading the OD values of the solution at (**a**) 585nm and (**b**) bacterial growth at 600 nm.The statistical significant of each test (n = 3) was evaluated by conducting one-way ANOVA test and a P value of P < 0.05 being significant. Inset: Structure of caffeine.

The violacein produced by treated *C. violaceum* CV026 overnight culture was extracted as described in materials and methods. Ultrapure water and 20% DMSO served as negative controls. The result was shown in Figure 3. Statistical test was also conducted using the ANOVA test and it was found that all tested caffeine concentrations showed a significant inhibition of violacein content (P < 0.05).

To confirm the anti-QS effect of caffeine was not due to any antibacterial activity, we monitored the bacterial growth at OD_600nm_. No significant antibacterial activity was shown by caffeine (P < 0.05) (Figure 3(b)). The result confirmed that caffeine at concentrations of 0.1, 0.2, and 0.3 mg/mL did not inhibit the growth of *C. violaceum* CV026. This confirmed that the reduction of the violacein production is due to anti-QS effects and not anti-bacterial properties.

### 3.4. Caffeine did not Degrade C6-HSL

**Figure 4.**
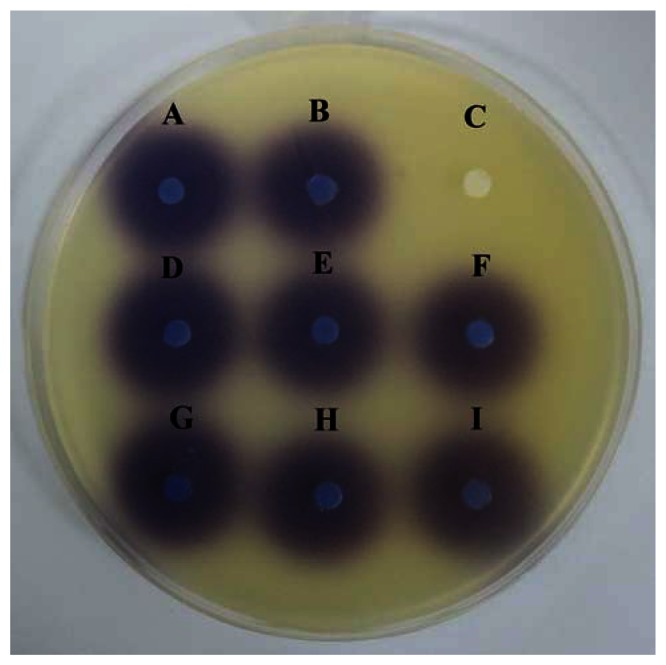
Caffeine did not degrade C6-HSL. Disc (**A**) C6-HSL treated with ultrapure water (negative control); (**B**) C6- HSL treated with 20% DMSO (negative control); (**C**) PBS; (**D**) C6-HSL treated with 0.1 mg/mL caffeine; (**E**) C6-HSL treated with 0.2 mg/mL caffeine; (**F**) C6-HSL treated with 0.3 mg/mL caffeine; (**G**) C6-HSL treated with 0.1 mg/mL (+)-catechin; (**H**) C6-HSL treated with 0.2 mg/mL (+)-catechin; (**I**) C6-HSL treated with 0.3 mg/mL (+)-catechin. The result showed that both caffeine and (+)-catechin have no effect on C6-HSL. This assay was conducted in three independent triplicates.

This assay was conducted to confirm the anti-QS property of caffeine were not due to the degradation of the C6-HSL. Figure 4 showed that caffeine and (+)-catechin at the tested concentrations did not degrade C6-HSL.

### 3.5. Caffeine Inhibited P. aeruginosa PA01 Short Chain AHLs Production

**Figure 5.**
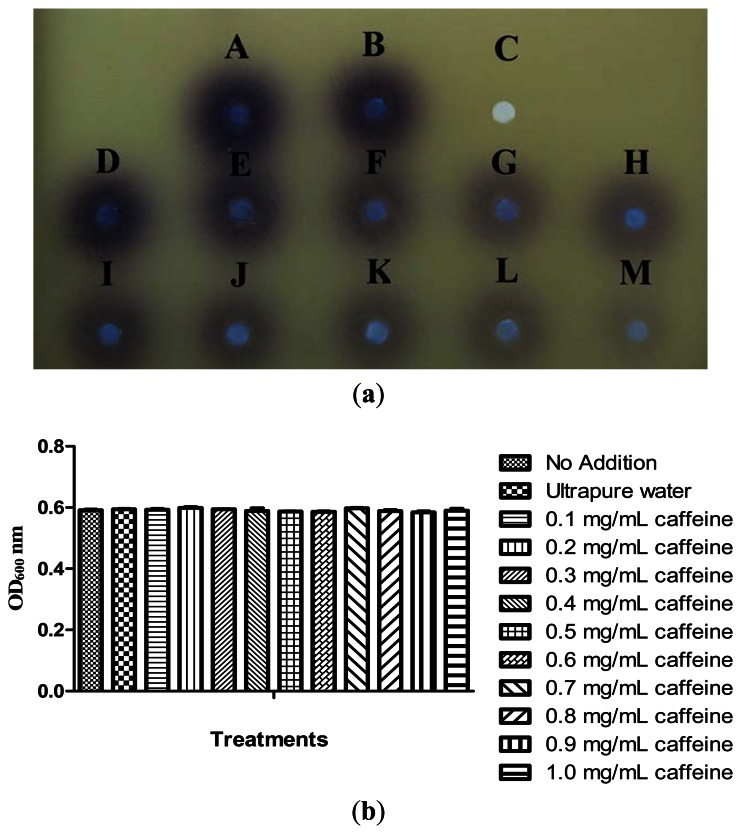
(**a**) Caffeine inhibited *P. aeruginosa* PA01 QS signal production. Filter paper A and B were added with 20 μL AHL extracted from *P. aeruginosa* PA01 culture (A); *P. aeruginosa* PA01 culture treated with ultrapure water (B). In (C) only PBS buffer (20 μL) was spotted on the CV026 lawn. Filter paper (D); (E); (F); (G); (H); (I); (J); (K); (L); and (M) were added with 20 μL AHLs extracted from *P. aeruginosa* PA01 culture treated with 0.1, 0.2, 0.3, 0.4, 0.5, 0.6, 0.7, 0.8, 0.9 and 1.0 mg/mL caffeine, respectively. This assay was conducted in three independent triplicates and representative data are shown. This result shows that caffeine interfered AHLs production of *P. aeruginosa* PA01 in a concentration dependent manner as evident. (**b**) OD_600nm_ reading of *P. aeruginosa* PA01 overnight culture treated with various concentrations of caffeine. The statistical significant of each test (n = 3) was evaluated by conducting one-way ANOVA test and a P value of P < 0.05 being significant. Our result indicated no significant differences between the treatments and the control at P < 0.05 suggesting that caffeine at these concentrations did not exhibit any antibacterial activity in *P. aeruginosa* PA01.

Our previous results have shown that caffeine did not degrade C6-HSL. We then further investigated the inhibition of *P. aeruginosa* PA01 AHL signal production by caffeine. It has been reported that *Pseudomonas* spp. triggered CV026 violacein production, suggesting production of short chain AHLs [30]. With this knowledge, a preliminary test was designated to test the inhibition of short chain AHLs produced by *P. aeruginosa* PA01 with various concentrations of caffeine ranging from 0.1 to 1.0 mg/mL. Our result shown a decreasing in the intensity of purple pigment (violacein) formed on the CV026 lawn is directly proportionate to the concentration of caffeine applied. When caffeine was applied at the concentration of 1.0 mg/mL, only trace amount of AHLs production by *P. aeruginosa* PA01 was observed (Figure 5(a)) and this is not due to antibacterial effects (Figure 5(b)).

To ensure caffeine (at various concentrations) showed anti-QS instead of antibacterial activity, we monitored the growth of *P. aeruginosa* PA01 and the data was analyzed using one-way ANOVA test. Our result showed no significant differences between the treatments and the control (P < 0.05). This indicated that caffeine at concentrations ranging from 0.1 to 1.0 mg/mL did not inhibit the growth of *P. aeruginosa* PA01 hence confirmed that the reduction of the AHLs production is due to the inhibition of *P. aeruginosa* PA01 short chain AHL production. Similar results were obtained when the growth of *P. aeruginosa* PA01 was further monitored for duration of 24 hours (data not shown).

### 3.6. Caffeine Inhibited Pseudomonas aeruginosa PA01 Swarming Motility

**Figure 6.**
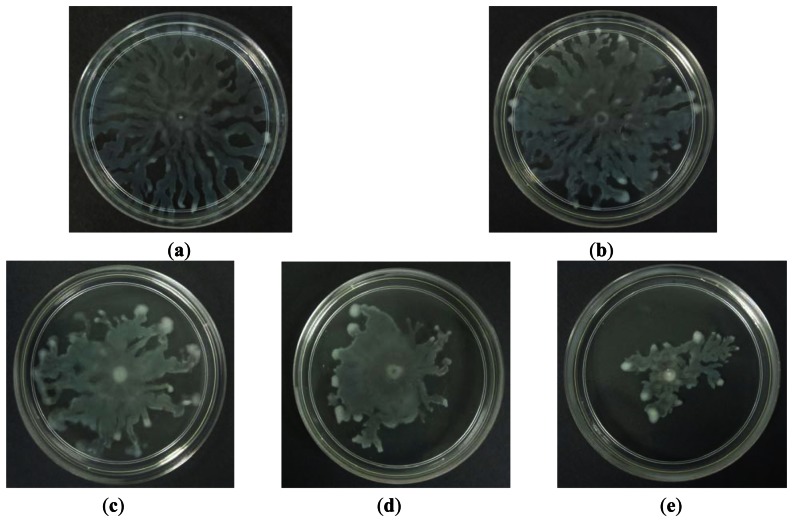
Swarming inhibition assays. Swarming agars of *P. aeruginosa* PA01 (**a**); supplemented with ultrapure water (**b**); (v/v, negative control); and caffeine of 0.1 mg/mL (**c**); 0.2 mg/mL (**d**); and 0.3 mg/mL (**e**). Images shown are *P. aeruginosa* PA01 swarming patterns and inhibition effects after 16 hours of incubation at 37 °C. This assay was conducted in three independent triplicates.

Swarming is a complex type motility that is defined by rapid and coordinate translocation of a bacterial population across a semi-solid surface [29]. A bacterial swarming motility was shown by the observation of long and hyperflagellated cells formed on the swarming plate. In addition to flagella and pili, swarming of *P. aeruginosa* also requires the production of two biosurfactants; rhamnolipids and 3-hydroxyalkonics acids [31]. Study conducted has found out that the production of rhamnolipids in *P. aeruginosa* PA01 is controlled by QS [32]. In this study we investigated the ability of caffeine to inhibit *P. aeruginosa* PA01 swarming activity. Caffeine was seeded into the swarming agar and ultra-pure water was used as negative control. Our results (Figure 6) indicated that caffeine at 0.3 mg/mL concentration showed observable inhibition against the swarming of *P. aeruginosa* PA01 with formation of bacterial colony of short and undefined tendrils.

## 4. Conclusions

In summary, our results have demonstrated caffeine as a potential QS inhibitor. We also verified that caffeine inhibited swarming and AHL production of *P. aeruginosa* PA01. It is confirmed that caffeine did not degrade AHLs, but rather inhibited its production. At this point, few questions remained to be answered for instance, can caffeine inhibit the AHL synthesis by changing the structures of the AHL synthase, or competitive and/or non-competitive binding to the AHL synthase and/or AHL receptors by blocking AHLs from forming AHL-receptor complex? Further work will have to be done to address these issues.
